# Enhanced Surveillance for Detection and Management of Infectious Diseases: Regional Collaboration in the Middle East

**DOI:** 10.3402/ehtj.v6i0.19955

**Published:** 2013-01-25

**Authors:** Alex Leventhal, Assad Ramlawi, Adel Belbiesi, Sami Sheikh, Akhtam Haddadin, Sari Husseini, Ziad Abdeen, Dani Cohen

**Affiliations:** 1 Ministry of Health, Israel; 2 Palestinian Ministry of Health, Ramallah, West Bank, Palestinian Authority; 3 Jordan Ministry of Health, Amman, Jordan; 4 Search for Common Ground, Jerusalem; 5 Al-Quds Nutrition and Health Research Institute, Faculty of Medicine, Al-Quds University, Jerusalem; 6 School of Public Health, Sackler Faculty of Medicine, Tel Aviv University; 7 Braun School of Public Health, Faculty of Medicine, Hebrew University, Jerusalem

**Keywords:** Middle East, cross-border collaboration, foodborne disease surveillance, avian influenza preparedness, pandemic influenza

## Abstract

Formed before international negotiations of the revised International Health Regulations (IHR), the Middle East Consortium for Infectious Disease Surveillance (MECIDS) is a regional collaboration aimed at facilitating implementation of the revised IHR and, more broadly, improving the detection and control of infectious disease outbreaks among neighboring countries in an area of continuous dispute. Initially focused on enhancing foodborne disease surveillance, MECIDS has expanded the scope of its work to also include avian and pandemic influenza and other emerging and re-emerging infectious diseases. Here, we describe the history and governance of MECIDS, highlighting key achievements over the consortium's seven-year history, and discuss the future of MECIDS.

## Introduction

Since the last decade of the previous century, there has been a continuous trend of increasing globalization of commerce, travel, production, and services. While this new phenomenon is providing many countries with significant economic advantages, it is also increasing the risk of novel infectious disease threats. Other environmental, host, and agent-related factors have contributed to the emergence or re-emergence of various infectious diseases (1, 2). The broad geographical dispersal of these newly emerging pathogens in both products and people has raised the need for new surveillance and response capabilities. The ability to sensitively, specifically, and promptly identify particular strains or subtypes of organisms using modern diagnostic techniques has become essential for rapidly and efficiently responding to disease outbreaks and preventing potential epidemic or pandemic spread. Additionally, as recent outbreaks of severe acute respiratory syndrome (SARS) and avian and pandemic influenza have demonstrated, these new threats are changing the way that outbreaks are dealt with—from mainly local and national responses to regional and even global approaches (2–4).

Global collaboration in infectious disease surveillance is orchestrated by the World Health Organization (WHO), most recently through the revised International Health Regulations (IHR 2005) (5). A legally binding document signed by all WHO Member States, the revised IHR set rules for improving communication between WHO and Member States and mandate that each country has the laboratory capacity to rapidly identify outbreaks. A year before the international negotiation of the revised IHR, a sub-regional surveillance network, the Middle East Consortium for Infectious Disease Surveillance (MECIDS), was established as a means to facilitate implementation of the IHR in three neighboring countries: Jordan, Israel, and the Palestinian Authority (PA). Since then, MECIDS has enjoyed a close relationship with the WHO, not just with respect to the revised IHR (see Text Box 1), but also with respect to receiving expert advice from WHO officials on any issue. Many MECIDS meetings are attended by officials from WHO headquarters in Geneva and from the WHO Eastern Mediterranean and European region offices. Although they are neighbors, the three countries extend across two different WHO regions: the Eastern Mediterranean and European regions. This paper reviews the development and deployment of this unique public health surveillance system.

***Text Box 1*. MECIDS and the International Health Regulations (IHR)**

Although MECIDS was established before the final resolution by consensus on the new version of IHR 2005 (5), it has a special relationship with the World Health Organization (WHO) in general and concerning the IHR in particular. This is true even of the PA, who is not a member party in the IHR agreement (i.e., because the PA is a member of MECIDS, Palestinian officials participate in all MECID activities). In addition to the WHO generously providing public health advisors for MECIDS projects. MECIDS principals were chief delegates of their countries for international negotiations around the revised IHR; and MECIDS was operating according to the principles of the revised IHR even before the IHR were implemented. For example, during the AI outbreaks in 2006 (see Case Study No. 2), MECIDS partners decided to act according to the revised IHR even though the regulations were not being enforced yet (13). In 2007, MECIDS partners participated in a special workshop on how to implement the IHR in the MECIDS region. WHO officials from the IHR headquarters in Geneva and from the Eastern Mediterranean and European Region offices attended the meeting. Most recently in June, 2012, senior WHO officials attended a MECIDS workshop where officials from all three MECIDS country ministries of health drafted a trilateral public health agreement for regional land border crossing. The following month, MECIDS shared their IHR experience at a WHO IHR seminar in Lyon, France.

## History and Governance

The potential for a Middle Eastern partnership in infectious disease surveillance was discussed in November, 2002, at a meeting held by two Washington, D.C.-based non-governmental organizations, Search for Common Ground (SFCG) and the Nuclear Threat Initiative (NTI). Meeting participants included public health officials and academics from Jordan (Ministry of Health and Royal Scientific Society), Israel (Ministry of Health, Tel Aviv University), and the Palestinian Authority (PA) (Ministry of Health, Al-Quds University). The vision was of a partnership that facilitates cross-border cooperation in public health, particularly in response to infectious disease outbreaks, through capacity building and also by encouraging human relationships that enhance regional stability and security.

The initial focus of MECIDS was on the sharing of data on foodborne disease outbreaks, specifically *Salmonella*. The same approach was more recently implemented for *Shigella*, another foodborne and person-to-person transmitted enteropathogens. Since then, data sharing has expanded to other disease areas, including avian and pandemic influenza and, most recently, vector-borne diseases.

Although its work began earlier (e.g., as described in Text Box 2, what would eventually become MECIDS held its first training in 2004), MECIDS was formally established in 2007. MECIDS maintains a secretariat headquarters at SFCG, Jerusalem, and is governed by an Executive Board that meets twice a year. Chairmanship of the Board rotates among countries every year, and Board decisions are reached by consensus. Since its formation, MECIDS has received contributions from various donors, including NTI; the World Bank (Washington, D.C.); Becton, Dickinson and Company (BD) (New Jersey, United States), and the International Council for the Life Sciences (ICLS) (Virginia, United States).

***Text Box 2*. Training**

Regional training is an ongoing MECIDS activity. Initially, trainings were aimed at improving *Salmonella* diagnostic capabilities. In September, 2004, 35 Palestinian, Jordanian, and Israeli health professionals participated in a five-day workshop in Istanbul on key epidemiology concepts and other relevant knowledge that would help the professionals to monitor and respond to regional disease outbreaks. The following spring, microbiologists from all three countries attended a *Salmonella* identification workshop in Israel, where they received hands-on training from specialists at the Jerusalem Central Laboratory of the Israeli Ministry of Health. The four-day curriculum covered a range of topics, including *Salmonella* serotyping and phage typing, pulsed field gel electrophoresis (PFGE), use of the Vitek machine that MECIDS purchased for all three countries, and antibiotic resistance testing. In April, 2008, an additional training course on interventional epidemiology was held in Israel; 33 professionals from Israel and the Palestinian Authority attended.

Recently, efforts have shifted away from Salmonella, toward other issues. Laboratory and public health professionals from all three countries have attended meetings dedicated to the identification and characterization of another foodborne pathogen, *Shigella*; general laboratory safety and security issues; and the use of bioinformatics in microbiology and molecular epidemiology.

## Activities and Achievements

MECIDS has been effective on many levels. In addition to sharing data and analyses, MECIDS partners have harmonized their infectious disease diagnostic and reporting methodologies; conducted joint trainings; and facilitated cross-border communication between laboratory technicians. Additionally, the partners established protocols for collaborative cross-border investigation of infectious disease outbreaks; set up an automatic notification system for cross-border events; and tested their preparedness for pandemic influenza. Here, we highlight three case studies that reflect the progress MECIDS has made during the last seven years.

### Case Study 1: Laboratory-Based Enhanced Foodborne Disease Surveillance System

**Fig. 1:**
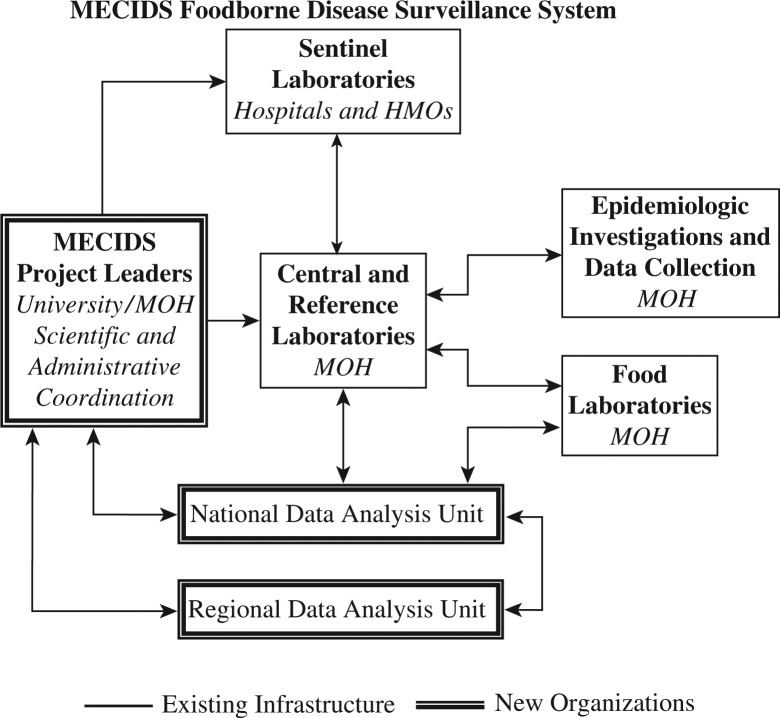
A schematic of the MECIDS foodborne disease surveillance network. Source: MECIDS.

The consortium's first major undertaking was to establish a regional laboratory-based foodborne disease surveillance network (Figure 1). MECIDS partners agreed that significant upgrading in foodborne disease surveillance methods would play an important role in preventing and controlling the emerging foodborne disease outbreaks, which public health experts were predicting would increase as food trade in the region increased. Also, of note, as part of the WHO strategy to reduce the global burden of foodborne diseases, Jordan had been selected as the first sentinel site in the WHO Eastern Mediterranean region for a series of studies on the burden of *Salmonella*, *Shigella*, and *Brucella* diseases. The studies revealed that foodborne disease burden was being underestimated and called for establishment and enhancement of sentinel laboratory-based surveillance for both *Salmonella* and *Shigella* in particular (6–8). Because of the likelihood that MECIDS would expand to other countries in the Middle East in the future and so that the network could be integrated with other existing networks in Europe (e.g., Enter-ne, Salm-Gene) and the United States (e.g., FoodNet, PulseNet), the partners decided to build a network that was comparable to those existing networks (9, 10).

Specifically, MECIDS chose *Salmonella* as its first foodborne pathogen target. The partners sought to establish a network of sentinel microbiological laboratories with the capabilities to identify *Salmonella*; harmonize data collection methodologies and built a common platform for communication, data sharing, and analysis; and strengthen reference laboratory capabilities to characterize *Salmonella* phenotypes (i.e., serotypes) and genotypic markers. In view of differences in existing capabilities and infrastructures between countries, the partners agreed that each country would outline its own specific immediate objectives which, once met, would help to achieve the overall goal of a regional foodborne diseases surveillance network (11). Each country selected which microbiological labs would serve as sentinel labs in the network; and designated a National Reference Lab (NRL).

In addition to selecting which laboratories would participate in the network, partners also developed standard testing procedures. The surveillance population was defined as patients attending sentinel labs for stool and/or blood cultures, food-handlers attending sentinel labs for stool cultures, and food items received by food labs. Specimens are tested for the presence of *Salmonella* using the same standard operating procedures; organisms identified as *Salmonella* in sentinel labs are submitted to the NRL for serogrouping, serotyping, and antimicrobial susceptibility tests. Also at the NRL, pulsed field gel electrophoresis (PFGE) is performed on selected isolates using standard protocols developed by the Salm-gene network in Europe. As a rule, *Salmonella* isolates are preserved at −70°C for further testing and genotyping.

Additionally, each country established a data analysis unit to manage all of the surveillance data and to serve as a central national focal point. Data collection started in 2005 (i.e., two years before MECIDS was formally established). Data include patient information (sex, age, if they are inpatient or outpatient subjects, address, etc.), as well as specimen type (stool, blood or urine) and isolate (*Salmonella* serogroup and serotype). In each country, data collected from both the sentinel labs and NRL are recorded in specifically designed data collection forms and sent on regular basis to the designated national data analysis units (i.e., the Disease Control Directorate in Jordan, Disease Control in the Palestinian Authority, and the Center for Diseases Control in Israel). When an outbreak is first detected, the national data analysis units play a major role in alerting the public health authorities and initiating epidemiological investigations (11).

The MECIDS consortium also identified a regional data analysis unit: the Middle East Scientific Institute for Security (MESIS), located in Amman, Jordan; and established a mechanism for the national systems to share their data with the regional unit. Country reports that have been prepared by the national data analysis units, excluding patients’ personal identifiers, are sent routinely to the regional unit where data are stored and regularly posted on the MECIDS website; national data are secured and only MECIDS members and authorized users are able to access them. Also, interim regional reports are presented and discussed at MECIDS executive board meetings; and posted on the consortium website. A manuscript compiling analyzed data of the first six years of regional *Salmonella* surveillance is in preparation.

Establishment of a Middle East regional laboratory-based foodborne disease surveillance network was a process – one that required building human and technical capacity so that partners could work together at similar levels of capability. This capacity was built largely through collaborative training. Text Box 2 describes a series of joint training courses on interventional epidemiology and laboratory technology that addressed not just *Salmonella* diagnostic capabilities, but also *Shigella* surveillance and regional infectious disease surveillance in general. In some cases, the necessary capacity building also involved the supply of equipment. For example, MECIDS developed support for the supply of PFGE equipment to Jordan and Palestinian Authority (both for the West Bank and Gaza), enabling both partners to collaborate with Israel which already possessed the equipment.

MECIDS researchers have also been involved in a variety of research projects on infectious disease burden in the Middle East. In 2011, MECIDS researchers reported on the underestimation of childhood diarrheal disease burden in Israel (12). More recently, MECIDS scientists completed a still unpublished study on the source and mode of transmission of *Salmonella* Infantis in Israel, where the proportion of *Salmonella* isolates identified as *S*. Infantis dramatically increased after 2009. Interestingly, recent serotyping of a large collection of *Salmonella* isolates from Jordan, Palestinian Authority and Israel showed high similarity in the distribution of *Salmonella* serotypes in Israel and the Palestinian Authority and differences in comparison to that of the Jordanian serotypes. These findings are most probably related to the closed links in food trade between Israel and Palestinian Authority.

### Case study 2: Response to Avian Influenza (AI) in 2006 (13)

**Fig. 2:**
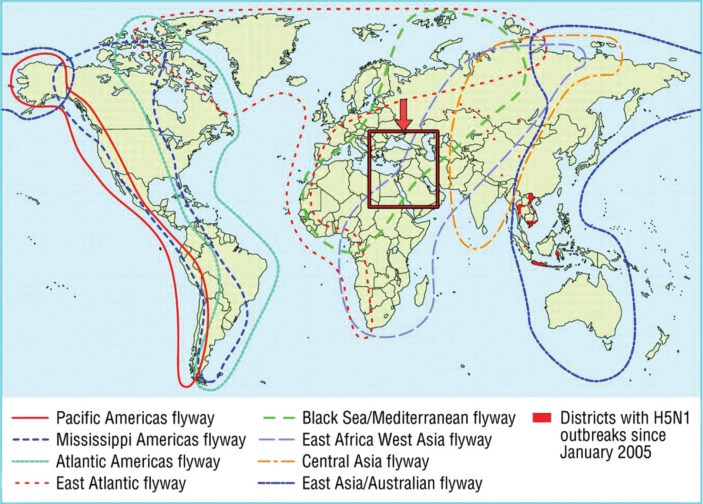
Major bird migratory pathways worldwide, with MECIDS countries (in the box) serving as a “bottleneck” for the Black Sea/Mediterranean and East Africa/West Asia flyways. Source: MECIDS.

MECIDS partners share a unique geographical situation. Located at the junction of three continents (Asia, Africa and Europe), between the Mediterranean Sea and Arabian Desert, the three countries act as a “bottleneck” through which a large portion of the world populations of certain migratory bird species concentrate on their way to and from their winter quarters in Africa (Figure 2). These birds serve as a continuous source of viruses, such as West Nile and avian influenza (AI). It has been estimated that every year approximately 500 million birds pass through Israel alone.

Thus, when the H5N1 AI pandemic threatened the Middle East, with poultry outbreaks occurring in nearby Turkey (October 2005), the Ministries of Health and Agriculture of the three MECIDS countries agreed to hold a meeting to discuss the threat. The meeting, which was held in Istanbul, Turkey, in December, 2005, was attended by senior officials from the Ministries of Health and Agriculture of Jordan, Israel, and the PA, as well as senior officials from the Egyptian Ministry of Health, WHO, U.S. Centers for Disease Control (CDC), and the European Union. Each country presented its national AI and pandemic influenza preparedness plan (the Ministries of Health of the attending countries had been drafting national preparedness plans since 2003 or before), and the foundation was set for real-time exchange of information in the event of an AI outbreak in the region.

Two months later, on February 16, 2006, another coordination meeting on AI took place on the King Hussein Bridge, a land crossing between Israel and Jordan, in order to share information on recent developments in AI preparedness. On the following day, the first AI outbreak in Egypt was detected involving backyard poultry, wild bird, and human cases. Although this outbreak took place hundreds of miles from the MECIDS countries, the threat was clearly imminent.

Indeed, on March 16, 2006, the first case of AI in any MECIDS country was diagnosed in Israel in some industrial coop turkey populations near the border with the PA Gaza Strip. The diagnosis was confirmed by the Israeli central veterinary laboratory. Four suspected human cases were referred to hospital emergency rooms, but none turned out positive for AI. This event was communicated immediately by phone to points of contact at the Palestinian and Jordanian Ministries of Health that had been designated at the Istanbul meetings. The World Organisation for Animal Health (OIE) and WHO were also immediately notified. Over the course of the next two weeks (March 16–31, 2006), a total of nine outbreaks of AI were recorded in industrial poultry coops across Israel. Five of these outbreaks were in coops bordering the Gaza Strip, of which one was in proximity to the Egyptian border; one outbreak took place in a poultry coop near Jerusalem, in proximity to the West Bank; and one outbreak took place in a settlement in the northern Jordan Valley near the border between Israel and Jordan. All poultry within three kilometer protective zones around each of the nine outbreak foci (i.e., a total of 1.2 million birds in Israel) were culled by poisoning their drinking water.

In addition to the AI outbreaks in Israel, samples from sick poultry in Gaza that were sent by the PA veterinary services to the Israeli central veterinary lab on March 22 2006, also tested positive for H5N1. In response, on that day, a meeting took place at the Gaza Crossing, with both Israeli and Palestinian veterinary and health officials attending. The officials agreed on shared protocols for coping with the outbreak and arranged for personal protective equipment, Oseltamivir tablets, and poison for poultry culling to be transferred from Israel to the PA. Over the course of the next two weeks, H5N1 virus was diagnosed in four additional locations along the Gaza Strip, among both industrial coop and backyard poultry populations. Authorities culled 600,000 birds using the same method that had been employed in Israel.

During the same time period, on 24 March 2006, Jordan reported an H5N1 outbreak in backyard turkeys in a Kofranja village (Ajloun) east of the Jordan Valley, 25 kilometers northeast of the previously mentioned infected Israeli coop in the Jordan Valley. The event was promptly reported to both Israeli and Palestinian “contact points” in the Health and Agriculture Ministries. Jordanian authorities culled 20,000 birds in the three kilometer protective zone.

On March 27, 2006, a tri-country coordination meeting took place in Jerusalem. The meeting was also attended by the WHO officer to the PA and a member of the Egyptian Embassy in Israel. Meeting attendees shared information regarding the regional AI threat and discussed cooperation, coordination and assistance among the Health and Agriculture Ministries.

Today, looking back six years after the event, MECIDS partners believe that the cooperation, mutual reporting and assistance that occurred at the time and which are described here significantly thwarted the AI threat. The opportunity to compare and synchronize preparedness plans prior to the event (i.e., during the Istanbul meeting in December, 2005) contributed to the successful mitigation and communication efforts that occurred during the actual AI outbreaks. The cooperation that occurred during the AI outbreaks extended beyond the neighboring countries providing each other with tangible aid (e.g. supplying the equipment necessary for bird culling). But also, public health officials in all three countries were updated in real-time. In addition, communication with the media was harmonized and contradictory messages to the public were prevented. The experience built trust and confidence among MECIDS member countries in cross-border health crisis management – a confidence that was tested and proven when the 2009 H1N1 pandemic influenza threatened the region.

### Case study 3: Regional Response to the 2009 H1N1 Influenza Pandemic (14)

Following the AI crisis, in 2007 and 2008 MECIDS conducted a series of national pandemic influenza tabletop exercises to identify gaps in preparedness and cross-sectoral cooperation and to develop a plan of priority actions to improve preparedness and response. Also in 2008, the partners conducted a regional tabletop exercise to test cross-border cooperation and procedures in the event of a pandemic. The regional exercise involved not just public health experts and ministry of health officials, but also representatives from the transportation, education, interior, laboratory, and media sectors. The exercise was conducted in cooperation with observers from WHO (from headquarters in Geneva and both the Eastern Mediterranean and European regional offices) and the Turkish Ministry of Health. The following year, a novel influenza virus, H1N1, began its global spread.

On April 27, 2009, just two days after WHO raised the H1N1 pandemic threat level to Phase 4, MECIDS partners held an emergency teleconference to discuss a joint plan of action to mitigate the spread of H1N1 into and out of the Middle East. At that time, there were a few suspected cases in Israel. Two days later, on April 29, in response to H1N1 influenza outbreaks throughout the world, WHO Director-General Dr. Margaret Chan raised the influenza pandemic alert from Phase 4 to Phase 5. On May 1, MECIDS partners met at the WHO office in East Jerusalem with observers from the WHO and the Egyptian Embassy. The partners agreed to implement and coordinate prompt border and airport screening, laboratory testing, information exchange, and common communication strategies. This coordination was made possible by the confidence in cross-border health crisis management and trust among MECIDS partners that had been building over the past several years and to well-exercised national and regional pandemic preparedness plans. The need for such coordination was made all the more critical by the coinciding detection of new cases of avian influenza H5N1 in Egypt and concerns that the two influenza viruses would combine and form a new pandemic influenza virus.

In mid-June, the WHO raised the influenza pandemic alert from phase 5 to phase 6. At about the same time, Jordan and the PA reported their first cases of H1N1, mostly among university students returning from summer vacations in Canada and the United States.

Not until July 16, when WHO acknowledged that further spread of the pandemic was inevitable and that individual case counting was no longer essential, did the three MECIDS countries stop sharing daily reports of new cases.

## Challenges Faced and Lessons Learned

The various national and regional networks of collaboration, communication, and information exchange that MECIDS partners have established over the past seven years are helping the partners not only estimate disease burden (9) but also harmonize public health intervention and prevention strategies (13, 14). The laboratory surveillance systems established or strengthened by MECIDS are an important component of this regional effort. However, a key challenge facing MECIDS is the significant lag time that still exists between the different stages of surveillance data collection and reporting (i.e., sentinel lab diagnosis, reference lab characterization of isolates, reporting). This time lag prevents real-time use of data. Another challenge is the need to be cognizant of variation in cultural and scientific sensitivities and representativeness that exists among the three MECIDS partner countries when making data comparisons at the regional level.

## Moving Forward: Sustainability of MECIDS

As demonstrated in Case Studies No. 2 and 3, the platform of collaboration that MECIDS has established since that first seminal meeting in November, 2002, has become sustainable – even during times of political dispute and outbreaks of violence, as occurred in the Middle East during the influenza outbreaks of 2006 and 2009.

The “bottom-up” evolution of MECIDS through interactions between public health officials on opposite sides of country borders has been an important driver of MECIDS's success. The consortium was not built through a “top-down” directive from member countries or from agencies outside the sub-region.

Indeed, MECIDS has become a good example for other infectious disease networks that have emerged over the years and, through Connecting Organizations for Regional Disease Surveillance (CORDS), is sharing its experience with others. At the same time, CORDS also enriches MECIDS with other networks’ experiences and good practices, especially with respect to implementing a “One Health” approach in tangible and rewarding ways and more than in the ad-hoc manner employed in response to the 2006 avian influenza outbreaks in MECIDS countries (Case Study 2).

MECIDS focus on foodborne diseases remains strong, with MECIDS partners not only responding to outbreak situations but also developing shared methods and a common regional database and researching the contribution of specific foods and foodborne pathogens to total disease burden. With more precise food- and pathogen-specific estimates, MECIDS partners will be able to construct effective food safety policies aimed at improving food trade and exchange in the region while simultaneously reducing the burden of foodborne disease. In the future, the consortium plans to extend its laboratory-based surveillance network from *Salmonella* and *Shigella* to other enteric pathogens, such as enterotoxigenic *Escherichia coli*, *Campylobacter jejuni*, and selected protozoa and viruses of public health importance in the Middle East.
